# Supplementary material for: QSM reconstruction challenge 2.0: A realistic in silico head phantom for MRI data simulation and evaluation of susceptibility mapping procedures
Source: Magn Reson Med. 2021 Feb 26;86(1):526–42. doi: 10.1002/mrm.28716 (PMC8048665; doi:10.1002/mrm.28716)
Supplement: Supplementary file 2 — TABLE S1 Ad hoc segmentation correction based on relaxometry values. Note: These corrections were only applied in regions where the magnetic field gradient, as computed from the multi‐echo data, was not expected to corrupt the R2∗ values [file MRM-86-526-s001.docx]

| Initial tissue segmentation | R1(mHz) criteria | R2*(mHz) criteria | Final tissue segmentation |
| --- | --- | --- | --- |
| CSF | -  R1>=250 | -  R2 > 70 | CSF  Blood |
| Grey matter | -  R1<330  R1>=330  R1 > 750 | -  -  R2 >= 100  R2 < 100 | Grey matter  CSF  Blood  WM |
| Caudate | -  R1<330  R1>=330  R1 > 750 | -  -  R2 >= 100  R2 < 100 | Caudate  CSF  Blood  WM |
| Putamen | -  R1<330  R1>=330  R1 > 750 | -  -  R2 >= 100  R2 < 100 | Putamen  CSF  Blood  WM |
| Thalamus | -  R1<330  R1>=330  R1 > 750 | -  -  R2 >= 100  R2 < 100 | Thalamus  CSF  Blood  WM |
| White matter | -  R1<330  R1>=330 | -  -  R2>100 | White matter  CSF  Blood |
| Blood (derived from Frangi filter maximum Diameter 6 voxels) |  | R2>75 | Blood |

Supplementary Information Table S1. Ad-hoc segmentation correction based on relaxometry values. Note that this corrections were only applied in regions where the magnetic field gradient, as computed from the multiecho data, was expected not to corrupt the R2* values
